# Astroglial tracer BU99008 detects multiple binding sites in Alzheimer’s disease brain

**DOI:** 10.1038/s41380-021-01101-5

**Published:** 2021-04-23

**Authors:** Amit Kumar, Niina A. Koistinen, Mona-Lisa Malarte, Inger Nennesmo, Martin Ingelsson, Bernardino Ghetti, Laetitia Lemoine, Agneta Nordberg

**Affiliations:** 1 Division of Clinical Geriatrics, Center for Alzheimer Research, Department of Neurobiology, Care Sciences and Society, Karolinska Institutet, Stockholm, Sweden; 2 Department of Physics and Astronomy, Uppsala University, Uppsala, Sweden; 3 Department of Pathology, Karolinska University Hospital, Stockholm, Sweden; 4 Department of Public Health and Caring Sciences, Geriatrics, Uppsala University, Uppsala, Sweden; 5 Department of Pathology and Laboratory Medicine, Indiana University School of Medicine, Indianapolis, IN USA; 6 Theme Aging, Karolinska University Hospital, Stockholm, Sweden

**Keywords:** Neuroscience, Diagnostic markers

## Abstract

With reactive astrogliosis being established as one of the hallmarks of Alzheimer’s disease (AD), there is high interest in developing novel positron emission tomography (PET) tracers to detect early astrocyte reactivity. BU99008, a novel astrocytic PET ligand targeting imidazoline-2 binding sites (I_2_BS) on astrocytes, might be a suitable candidate. Here we demonstrate for the first time that BU99008 could visualise reactive astrogliosis in postmortem AD brains and propose a multiple binding site [*Super-high-affinity* (SH), *High-affinity* (HA) and *Low-affinity* (LA)] model for BU99008, I_2_BS specific ligands (2-BFI and BU224) and deprenyl in AD and control (CN) brains. The proportion (%) and affinities of these sites varied significantly between the BU99008, 2-BFI, BU224 and deprenyl in AD and CN brains. Regional binding studies demonstrated significantly higher ^3^H-BU99008 binding in AD brain regions compared to CN. Comparative autoradiography studies reinforced these findings, showing higher specific binding for ^3^H-BU99008 than ^3^H-Deprenyl in sporadic AD brain compared to CN, implying that they might have different targets. The data clearly shows that BU99008 could detect I_2_BS expressing reactive astrocytes with good selectivity and specificity and hence be a potential attractive clinical astrocytic PET tracer for gaining further insight into the role of reactive astrogliosis in AD.

## Introduction

Amyloid plaques, neurofibrillary tangles but also neuroinflammatory markers such as aberrant astrocytes and microglia are considered to play an important role in the pathophysiological processes characterising the development of Alzheimer’s disease (AD). Astrocytes represent the 20–40% population of glial subtype-cells in the CNS and perform a wide array of functions needed for an optimal brain functioning and homeostasis [1–5]. In general, the human brain has an average of 50% of glial cells, but in the cerebral cortex, they account for 80% of the cells, whereas in the cerebellum, glia cells account for around 20% [6]. Moreover, they play a crucial role in regulating homeostasis of glutamate and γ-aminobutyric acid (GABA) transport through glutamate/GABA-glutamine shuttle [4, 7, 8] and can also induce synaptic plasticity and boost memory formation, as recently demonstrated [9, 10]. Most importantly, they respond to CNS insults/injuries and disease state by a specific defense process called reactive astrogliosis [11, 12]. According to a newly published consensus statement, reactive astrogliosis should be defined as the process where astrocytes engage in molecularly defined programs involving changes in transcriptional regulation, as well as biochemical, morphological, metabolic, and physiological remodeling, which ultimately result in gain of new function(s) or loss or upregulation of homeostatic ones [13]. Several recent studies have implicated reactive astrocytes mediated neuroinflammation in the etiology of AD and other neurological disorders [7, 14–17]. Postmortem studies have shown that reactive astrocytes are closely associated with amyloid-β plaques and tau (neurofibrillary tangles) in the cortical and hippocampal regions of AD brain [18–21]. Moreover, reactive astrocytes can undergo morphological changes and upregulate the production of glial fibrillary acidic proteins (GFAP) [11] and promote the release of inflammatory mediators such as cytokines (interleukin (IL)-1b and IL-6) and tumour necrosis factor-alpha [22], thereby triggering/promoting neuroinflammation as observed in AD [16, 17, 23, 24].

With recently published studies supporting the notion that reactive astrogliosis-mediated-neuroinflammation could precede other pathological hallmarks of AD during the early-stages of disease progression [15, 16, 25–29], there is a great need of developing novel astrocytic PET-tracers to monitor the function and in vivo distribution of astrocytes. So far only few PET-tracers targeting astrogliosis have been tested including ^11^C-deutrium-L-deprenyl (^11^C-DED), which targets the monoamine oxidase B (MAO B) enzyme [30, 31]. ^11^C-DED PET studies have been shown to be useful for visualising reactive astrogliosis in several chronic brain diseases such as epilepsy, Creutzfeldt-Jakob disease and amyotrophic lateral sclerosis [32–35].

We have previously demonstrated a high degree of reactive astrogliosis (^11^C-DED PET) in patients with mild cognitive impairment (MCI; amyloid positive [27] as well as in pre-symptomatic autosomal-dominant Alzheimer’s disease (ADAD) cases [36, 37]. We showed a negative correlation between reactive astrogliosis and amyloid plaque load and a positive correlation between ^11^C-DED and ^18^F-deoxyglucose uptake in ADAD cases suggesting that reactive astrogliosis is implicated in the initial stages of AD progression [37, 38]. Our immunostaining and autoradiography studies using ^3^H-Deprenyl have highlighted tight interconnection between tau pathology and reactive astrogliosis by demonstrating similar laminar cortical distribution for AT8 (tau) and GFAP antibody staining as well as for ^3^H-THK5117 and ^3^H-DED radiotracers in AD cases [39], including carriers of Arctic mutation in the amyloid-β precursor protein (*AβPParc*) [40]. These studies suggested good reliability of ^11^C-DED as an astrocytic tracer, however, there is still a high interest in developing more specific astrocytic PET-tracers and markers for reactive astrogliosis as MAO B expression is not limited to reactive astrocytes but is also observed in non-reactive astrocytes and in serotonergic, histaminergic [41] and cholinergic [42] neurons.

Tyacke et al. identified a new PET-tracer called ^11^C-BU99008, specifically and selectively targeting outer mitochondrial imidazoline_2_ binding sites (I_2_BS) that are predominantly expressed in astrocytes but also localised in neurons, albeit in a much lower density [43–46]. It is important to consider that the properties of I_2_BS can vary at molecular level between astrocytes and neurons as they represent a group of biologically diverse heterogenous proteins (molecular weight ~30, ~45–55, ~66, ~85 kDa) and the expression level of these receptor proteins can vary depending on the pathology [44]. Previous studies have shown a parallel link between I_2_BS and GFAP levels in AD brain [47] and have suggested a direct physiological role for I_2_BS in the regulation of GFAP expressions [48]. Upregulation of I_2_BS density has been seen in psychiatry disorders such as depression [49]. Most importantly, Ruiz et al. already in 1993 demonstrated by using mixed I_2_BS/α2-adrenoceptor inhibitor ^3^H-idazoxan a 63% increase in I_2_BS density in AD postmortem brain tissues [50], which further indicated that it could serve as an reliable surrogate marker to specifically visualise and monitor astrocyte (glial) activation in the human brain during normal and disease states.

Even though the animal preclinical studies [45, 46, 51] and the first human ^11^C-BU99008 PET-study in healthy volunteers demonstrated high specificity and selectivity for I_2_BS, good brain penetration and biodistribution for the tracer [43], no binding/comparative studies for BU99008 in AD cases are available so far to the best of our knowledge. The aim of this study was therefore to evaluate and compare the binding behaviour of the I_2_BS astrocytic PET tracer BU99008 in postmortem control (CN) and AD brain tissue using in vitro binding studies in order to estimate its potential as a novel astrocytic PET tracer for future clinical use and to compare it with I_2_BS-specific ligands (2-BFI and BU224) and the established astrocytic PET tracer deprenyl. We also aimed to compare ^3^H-BU99008 and ^3^H-Deprenyl binding behaviour in CN and sporadic AD tissue from large frozen human brain sections in a head-to-head in vitro autoradiography analysis.

## Materials and methods

### Chemicals

^3^H-BU99008 (specific activity (SA) = 83 Ci/mmol), ^3^H-L-Deprenyl (SA = 84 Ci/mmol) and unlabelled BU99008 were custom synthesised by Novandi Chemistry AB (Södertälje, Sweden). Unlabelled (R)-(-)-Deprenyl was purchased from Tocris Bioscience. The I_2_BS specific ligands 2-(4,5-Dihydroimidazol-2-yl)quinoline hydrochloride (BU224) and 2-(Benzofuran-2-yl)-2-imidazoline hydrochloride (2-BFI), the reversible MAO B inhibitor safinamide and all other chemicals (sodium chloride (NaCl), potassium chloride (KCl), calcium chloride (CaCl_2_), Tris base, magnesium chloride (MgCl_2_), disodium phosphate (Na_2_HPO_4_) and potassium dihydrogen phosphate (KH_2_PO_4_)) were purchased from Sigma-Aldrich AB, Sweden.

### Autopsy material

Human frozen brain tissue from six AD patients and seven CNs was provided by the Netherlands Brain Bank, Amsterdam, Netherlands (see Table 1. for clinical demographic data). The brain homogenates were prepared in 1X PBS buffer (pH 7.4) containing 0.1% BSA and protease/phosphatase inhibitors and were stored at −80 °C in aliquotes until use in binding experiments.

**Table 1:** Clinical demographic data for subjects used in this study.

	Sex (M/F)	Age (years)	Braak stage	ApoE (E/E)	Onset	Postmortem delay (hr:minutes)
*For binding studies*						
Control	F	50	1	3/3	N/A	4:10
	M	62	1	3/3	N/A	7:20
	F	71	1	3/2	N/A	7:10
	F	77	1	3/3	N/A	2:55
	M	78	1	3/3	N/A	<17:40
	M	79	2	3/3	N/A	9:00
	F	84	1	3/3	N/A	6:55
	**3M/4F**	**71.5** ± **11.8**	**1–2**	**7 E3 0 E4**		**7.7** ± **4.7**
Alzheimer’s disease	F	59	5	4/4	EOAD	4:20
	F	66	5	4/3	EOAD	6:30
	M	70	4	4/4	EOAD	4:00
	M	78	5	4/4	LOAD	6:35
	F	81	5	4/3	LOAD	6:15
	F	85	4	3/3	LOAD	6:00
	**2M/4F**	**73.1** ± **9.8**	**4–5**	**1 E3 5 E4**	**3EOAD/3LOAD**	**5.5** ± **1.0**
*For large frozen section autoradiography studies*						
Control	F	56	N/A	N/A	N/A	N/A
Sporadic Alzheimer’s disease^a^	F	79	V	4/4	LOAD	16:00
AβPPArc^a^	M	64	VI	3/3	EOAD	12:00

Bold values show the total number of male and female cases used in the study from control and AD cases as well as the means ± standard deviation of age, Braak stage, ApoE allele forms, and postmortem delay time from these cases.

*AβPPArc* Arctic amyloid-β protein precursor mutation, *ApoE* Apolipoprotein E, *M* Male, *F* Female, *EOAD* Early onset alzheimer’s disease, *LOAD* Late onset alzheimer’s disease, *N/A* Not applicable/available.

^a^Sporadic Alzheimer’s disease and AβPPArc cases have been also reported and described in our previous publications [39, 40, 70].

Large frozen whole right hemisphere brain tissue from one CN patient was provided by the Neuropathology of Dementia Laboratory, Indiana University School of Medicine, Indianapolis, IN, USA. Large frozen left hemisphere brain tissue from one patient with sporadic AD was provided by the Brain Bank at Karolinska Institutet and a large frozen left hemisphere brain tissue from one AβPParc carrier was provided by the Uppsala University brain bank. Refer to Table 1. for clinical demographic data of these cases. Direct comparisons between the cases should be taken with caution due to the fact that the large frozen sections were not from exactly the same coronal anatomical level.

### Saturation binding assays

The saturation binding assay was performed by incubating postmortem temporal cortex brain homogenate (0.1 mg tissue) from one CN and AD patient with increasing concentration of ^3^H-BU99008 (0–35 nM) in 50 mM Tris-HCl binding buffer pH 7.4 (50 mM Tris-base, 140 mM NaCl, 1.5 mM MgCl_2_, 5 mM KCl, 1.5 mM CaCl_2_) at 37 °C for 1 h. Non-specific (NSP) binding was determined with 1 μM unlabelled BU99008. The binding reaction was terminated by filtering through glass fiber filters (pre-soaked for 2–3 h in 0.3% polyethylenimine), followed by three quick rinses with cold binding buffer and overnight incubation of the filter in the scintillation liquid. The radioactivity in the tubes with reaction filters was counted on the next day with a beta scintillation counter (PerkinElmer Tri-Carb 2910TR). The saturation data was fitted and analysed using the non-linear regression function of GraphPad Prism 8.3 software [52] to calculate the dissociation constant (Kd) and maximum number of binding sites (Bmax). Scatchard plots were prepared with GraphPad Prism 8.3 software to display the saturation binding data. For the CN, the second binding site was drawn manually after fitting the data using the Hill slope-specific binding function in GraphPad Prism 8.3 software.

### Competition binding assays

Competition binding assays for ^3^H-BU99008 and ^3^H-Deprenyl were performed by using postmortem temporal cortex brain homogenate (0.1 mg tissue) from three CNs and two AD patients. The brain tissue homogenate was incubated with a single concentration of ^3^H-BU99008 (1 nM) and ^3^H-Deprenyl (10 nM) along with increasing concentrations of unlabelled BU99008 (10^−14^–10^−5^), 2-BFI (10^−11^–10^−5^), BU224 (10^−11^–10^−5^) and Deprenyl (10^−14^ or 10^−11^ to 10^−5^) in binding buffer (50 mM Tris-HCl buffer, pH 7.4 for ^3^H-BU99008 and 50 mM Na-K phosphate buffer, pH 7.4 for ^3^H-Deprenyl) for 1 h at 37 °C. After 1 h incubation a similar protocol as saturation binding assay was done and the binding was quantified using the scintillation counter.

^3^H-BU99008 competition binding assay with unlabelled BU99008 in the presence of reversible and irreversible MAO B inhibitors safinamide and deprenyl, respectively, was performed by using postmortem temporal cortex brain homogenate (0.1 mg tissue) from one CN and one AD patient. The brain tissue homogenate was incubated with a single concentration of ^3^H-BU99008 (1 nM) along with increasing concentrations of unlabelled BU99008 (10^−14^–10^−4^), BU99008 and Deprenyl (10^−14^–10^−4^) and BU99008 and safinamide (10^−14^–10^−4^) in binding buffer (50 mM Tris-HCl buffer, pH 7.4) for 1 h at 37 °C. A similar protocol as saturation binding assay was performed after 1 h incubation and the binding was quantified using the scintillation counter.

The competition binding data was fitted and analysed using non-linear regression competitive-binding function of GraphPad Prism 8.3 software [52] to determine IC50 (half-maximal inhibitory concentration) values.

### ^3^H-BU99008 brain regional binding studies

Regional binding studies were performed on the frontal, parietal and temporal cortices, cerebellum and hippocampus brain homogenates (0.1 mg tissue) from six CNs and six AD patients. Brain homogenates from different brain regions were incubated with a single concentration of ^3^H-BU99008 (1 nM) for 1 h at 37 °C in 50 mM Tris- HCI buffer, pH 7.4. After 1 h incubation, a similar protocol as saturation binding assay was done and the binding was quantified using the scintillation counter. Non-specific (NSP) binding was determined with 1 μM unlabelled BU99008. Cerebellum tissue from one CN, and frontal and hippocampal tissues from one AD case were not available. The binding data was analysed using GraphPad Prism 8.3 software [52] and presented in graphs as specific binding (fmol/mg).

### In vitro autoradiography studies

^3^H-BU99008 and ^3^H-Deprenyl autoradiography studies were performed on large frozen postmortem brain sections from one CN, one patient with sporadic AD and one *AβPPArc* carrier case. The large frozen sections were allowed to dry at room temperature (RT) for 30–45 mins, followed by 1 h incubation with either ^3^H-BU99008 (1 nM) or ^3^H-Deprenyl (10 nM) at RT. Afterwards, the sections were rinsed for 5 mins three times in cold buffer (50 mM Tris-HCl buffer, pH 7.4 for ^3^H-BU99008; 50 mM Na-K phosphate buffer, pH 7.4 for ^3^H-Deprenyl), followed by a quick dip in cold distilled water. The sections were allowed to dry at RT for 24 h and were then apposed together with a tritium standard (Larodan Fine Chemicals AB, Mälmo, Sweden) on a phosphor-plate for 4 and 7 days for ^3^H-Deprenyl and ^3^H-BU99008, respectively, and then imaged using a BAS-2500 phosphor imager (Fujifilm, Tokyo, Japan). For semiquantitative analyses, the regions of interest (ROI) were drawn manually using multigauge software on the autoradiogram and the photostimulated luminescence per square millimeter (PSL/mm2) was transformed into fmol/mg using the standard curve to determine the total, NSP and specific binding of ^3^H-BU99008 and ^3^H-Deprenyl in the ROI.

### Immunostaining on small paraffin sections

GFAP immunostaining (astrocytes) was performed on small paraffin section from the right hemisphere of sporadic AD brain using routine pathology method as described in our previous publication [39].

### Statistical analysis

The difference in regional ^3^H-BU99008 binding between CN and AD cases was statistically examined using Two-way ANOVA (with Sidak’s or Tukey’s multiple comparisons test) analysis in GraphPad Prism 8.3 software. An ANOVA *p value* < 0.05 was considered significant. The data are presented as mean of three experiments performed in triplicate with a box and whiskers plot.

## Results

### ^3^H-BU99008 saturation binding studies in CN and AD brain tissue

**Fig. 1:**
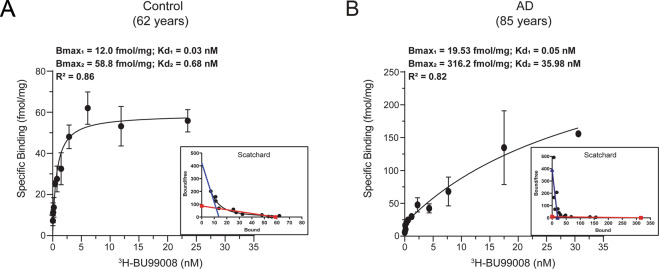
^3^H-BU99008 saturation binding assay. ^3^H-BU99008 saturation binding studies were performed in temporal cortex brain tissue homogenates from one CN and one patient with AD using increasing concentrations of ^3^H-BU99008 (0–35 nM). **A** and **B** show the saturation binding curves for the CN (62 years) and Alzheimer’s disease (85 years) cases, respectively, along with the corresponding Scatchard plots. For the CN, the second binding site was drawn manually after fitting the data using the Hill slope-specific binding function in GraphPad Prism 8.3 software (**A;** ***blue dotted line***). Data are presented as means ± SEM of two experiments in triplicate. *Kd- dissociation constant; Bmax- density of binding sites*.

The ^3^H-BU99008 saturation binding study results and the saturation curves and Scatchard plots for the CN and AD brain tissue are illustrated in Fig. 1. For the CN tissue, saturation occurred at a Kd_2_ of 0.68 nM and a Bmax_2_ of 58.8 fmol/mg (Fig. 1A). A second binding site (with a Kd_1_ of 0.03 nM and a Bmax_1_ of 12 fmol/mg) was also observed for the CN tissue when the Scatchard plot was manually drawn (Fig. 1A; blue dotted line). The saturation binding curve for the AD brain tissue showed two-binding sites with Kd_1_ and Kd_2_ values of 0.05 and 35.98 nm and Bmax_1_ and Bmax_2_ values of 19.53 and 316.2 fmol/mg, respectively (Fig. 1B), indicating a 60-fold lower affinity and 5-fold higher Bmax for ^3^H-BU99008 in AD brain tissue than in CN tissue for the low-affinity site.

### ^3^H-BU99008 competition binding studies with different unlabelled I_2_BS ligands in CN and AD brain tissue

**Fig. 2:**
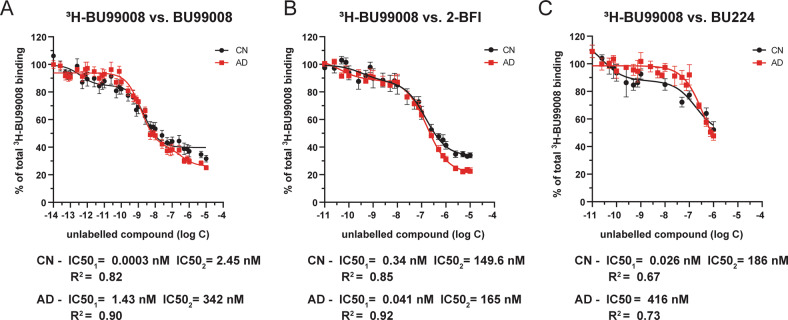
^3^H-BU99008 competition binding assay with different unlabelled I_2_BS ligands in CN and AD brain tissues. ^3^H-BU99008 competition binding studies were performed in temporal cortex brain homogenates from CN and AD brains using a single concentration of ^3^H-BU99008 (1 nM) and increasing concentrations of unlabelled I_2_BS ligands BU99008 (**A**), 2-BFI (**B**) and BU224 (**C**). Data are presented as means ± SEM of 4–7 experiments in triplicate using two tissue samples from two CNs (62 and 71 years) and two AD patients (78 and 85 years) for unlabelled BU99008 and 2-BFI, and one tissue sample from CN (50 years) and one AD patient (85 years) for unlabelled BU224. *2-BFI* = *2-(benzofuran-2-yl)-2-imidazoline hydrochloride, BU224* = *2-(4,5-dihydroimidazol-2-yl) quinoline hydrochloride, I*_*2*_*BS* = *imidazoline-2 binding sites, IC50- half-maximal inhibitory concentration*.

**Fig. 3:**
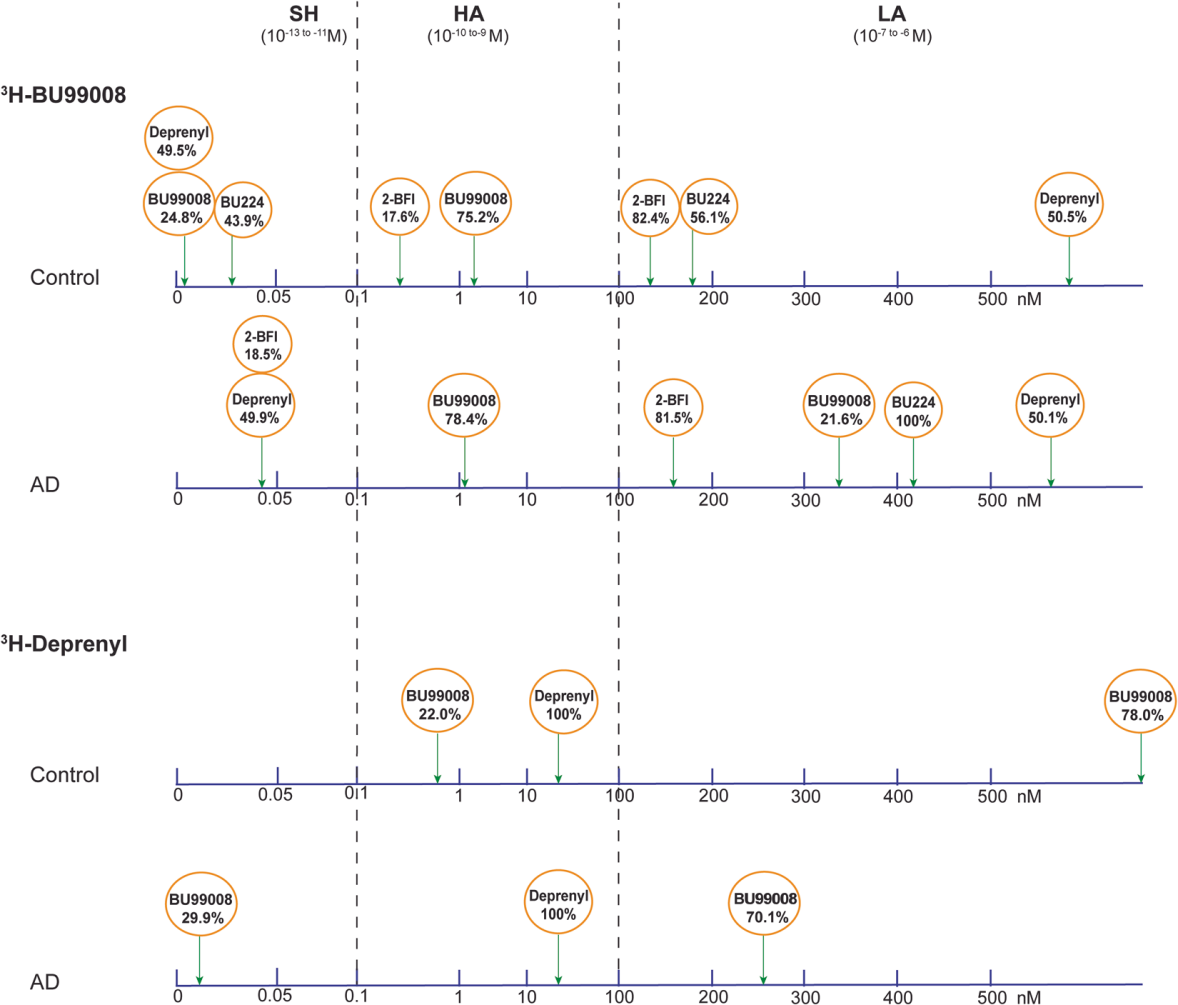
Proportions (%) of different binding sites for unlabelled BU99008, 2-BFI, BU-224 and deprenyl in postmortem CN and AD brain tissue revealed during ^3^H-BU99008 and ^3^H-Deprenyl competition studies. *SH- Super high-affinity binding sites, HA- high-affinity binding sites, LA- low-affinity binding sites*.

To further characterise ^3^H-BU99008 tracer binding properties, competition binding studies were performed with different concentrations of the unlabelled I_2_BS specific ligands BU99008, 2-BFI and BU224. The results of the competition studies suggested a range of binding sites with different affinities for ^3^H-BU99008 as shown in Fig. 2. Unlabelled BU99008 and 2-BFI both showed two-binding sites for both CN and AD cases (Fig. 2A and B), whereas unlabelled BU224 also showed two-binding sites for the CN and a single binding site for the AD (Fig. 2C). For unlabelled BU99008 and 2-BFI, the IC50_1_ values were 0.0003 nM and 0.34 nM for CN cases, respectively, and 1.43 and 0.041 nM for AD cases, respectively, while the IC50_2_ values were 2.45 nM and 149.6 nM for CN cases, respectively, and 342 nM and 165 nM for AD cases, respectively (Fig. 2A and B). In case of unlabelled BU224, the IC50_1_ and IC50_2_ values were 0.026 nM and 186 nM for the CN case, respectively, whereas the IC50 value for AD case was 416 nM (Fig. 2C). We further analysed the data by calculating the proportion (%) of different binding affinity sites for unlabelled BU99008, 2-BFI, and BU224 in CN and AD cases and grouped them into three groups (based on their IC50 values; Fig. 3): *Super high-affinity* (SH; 10^−13 to −11^ M), *High-affinity* (HA; 10^−10 to −9^ M) and *Low-affinity* (LA; 10^−7 to −6^ M) *binding sites*. The proportion of SH binding sites were 25% and 44% for BU99008 and BU224 in CN cases, respectively. Interestingly, BU99008 showed similar binding to HA binding sites for both CN and AD cases (75% and 78%, respectively) while a shift from SH binding sites (25%) in CN to LA binding sites (22%) in Alzheimer disease cases was observed. In CN, 2-BFI also showed a small percentage of HA binding sites (18%) while a majority of the binding to LA sites (82%). However, the LA binding sites remained the same in AD while the HA sites were shifted to SH binding sites (19%). BU224 showed approximate similar proportion of binding to SH (44%) and LA (56%) sites in CNs which were all shifted to LA binding sites (100%) in AD cases.

### ^3^H-BU99008 and ^3^H-Deprenyl comparative competition binding studies in CN and AD brain tissue

**Fig. 4:**
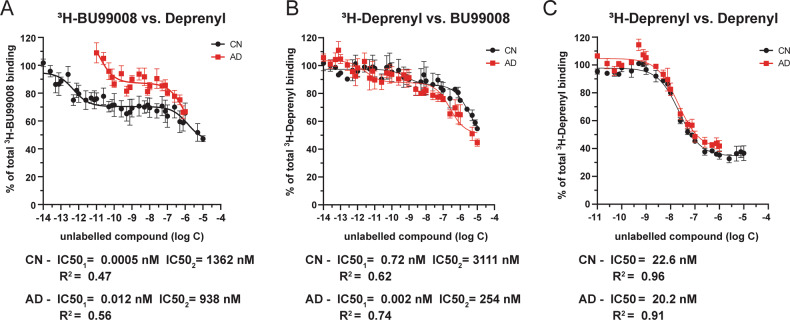
Comparison of ^3^H-BU99008 and ^3^H-Deprenyl competition binding assay with unlabelled deprenyl and BU99008 in CN and AD brain tissue. ^3^H-BU99008 and ^3^H-Deprenyl competition binding studies were performed in temporal cortex brain homogenates from one CN and one AD patient using a single concentration of ^3^H-BU99008 (1 nM) and ^3^H-Deprenyl (10 nM) against increasing concentrations of unlabelled deprenyl and BU99008: (**A**) ^3^H-BU99008 vs. deprenyl; **B** ^3^H-Deprenyl vs. BU99008; and **C** ^3^H-Deprenyl vs. deprenyl. Data are presented as means ± SEM of 3–4 experiments in triplicate using one tissue samples from CN (50 years for ^3^H BU99008 vs. Deprenyl; 62 years for ^3^H-Deprenyl vs. BU99008 vs. Deprenyl) and AD (85 years) cases for unlabelled BU99008 and deprenyl, respectively. *IC50- half-maximal inhibitory concentration*.

Next, we investigated the similarities/differences in the binding behaviour of ^3^H-BU99008 and ^3^H-Deprenyl with competition binding studies. We observed two-binding sites for unlabelled deprenyl vs. ^3^H-BU99008 while one-binding site vs. ^3^H-Deprenyl in both CN and AD cases, respectively (Fig. 4A and C). For unlabelled deprenyl and ^3^H-BU99008, the IC50 values were 0.0005 nM and 1362 nM for CN cases, respectively, and 0.012 and 938 nM for AD cases, respectively, while the corresponding IC50 values for deprenyl vs. ^3^H-Deprenyl were 22.6 nM for CN cases and 20.2 nM for AD cases (Fig. 4A and C). There were two-binding sites for ^3^H-Deprenyl vs. unlabelled BU99008 in both CN (IC50_1_: 0.72 nM, IC50_2_: 3111 nM) and AD cases (IC50_1_: 0.002 nM, IC50_2_: 254 nM) (Fig. 4B). For ^3^H-BU99008, unlabelled deprenyl thus showed almost identical proportion of binding to SH (~50%) and LA (50%) binding sites in both CN and AD cases, respectively (Fig. 3). On the other hand, for ^3^H-Deprenyl, unlabelled deprenyl showed 100% binding to HA site while unlabelled BU99008 demonstrated majority of binding to LA sites (~70–78%) in both CN and AD cases (Fig. 3).

### ^3^H-BU99008 competition binding studies with unlabelled BU99008 in the presence of two MAO B inhibitors in CN and AD brain tissue

**Fig. 5:**
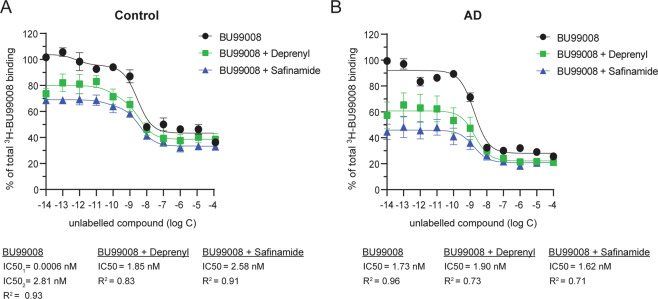
^3^H-BU99008 competition binding studies with unlabelled BU99008 in the presence of reversible and irreversible MAO B inhibitors. ^3^H-BU99008 competition binding studies with unlabelled BU99008 in the presence of MAO B inhibitors safinamide (reversible) or deprenyl (irreversible) were performed in temporal cortex brain homogenates from one CN (**A**; 71 years) and AD (**B**; 85 years) cases using a single concentration of ^3^H-BU99008 (1 nM) against increasing concentrations of unlabelled BU99008, BU99008 + Deprenyl or BU99008 + Safinamide, respectively. Data are presented as means ± SEM of three experiments in triplicate. *IC50- half-maximal inhibitory concentration*.

To further investigate the specificity of ^3^H-BU99008 for the HA binding site competition studies were performed with unlabelled BU99008 alone and in the presence of safinamide (reversible MAO B inhibitors) or deprenyl (irreversible MAO B inhibitors). For CN, unlabelled BU99008 alone showed two-binding sites with almost comparable IC50 values of 0.0006 nM (SH) and 2.81 nM (HA) as shown in Fig. 2 competition studies (compare Fig. 2A and Fig. 5A). Interestingly, in the presence of MAO B inhibitors deprenyl or safinamide, the displacement curves shifted towards right and showed monophasic behaviour with only a single site fit with IC50 values in the HA range (Fig. 5A). The IC50 values were 1.85 nM and 2.58 nM in the presence of deprenyl and safinamide, respectively. In addition, we also observed a decrease of ~15–30% in total binding in the lower concentration ranges [10^–14^–10^–9^] in the presence of deprenyl or safinamide (Fig. 5A). Whereas in case of AD the unlabelled BU99008 alone and in the presence of MAO B inhibitors showed almost similar one-site binding curves with comparable IC50 values in the HA range (Fig. 5B). The IC50 values were 1.73 nM, 1.90 nM and 1.62 nM for unlabelled BU99008 alone and in the presence of deprenyl and safinamide, respectively. Even though the curve for unlabelled BU99008 alone looked very similar as our competition studies (compare Fig. 2A and Fig. 5B), the LA binding site was undetectable in the analyses; this could be due to the smaller number of concentration points used in this experiment. Regardless of this, we also observed a pattern similar to that seen with the CN in the lower concentration ranges, with a decrease of ~30–55% in total binding in the presence of MAO B inhibitors (Fig. 5B).

### ^3^H-BU99008 regional distribution binding studies in CN and AD brain tissue homogenates

**Fig. 6:**
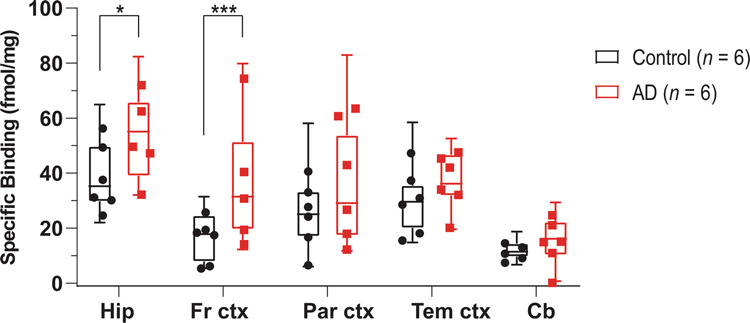
^3^H-BU99008 regional distribution binding studies in brain homogenates from CNs and patient with AD. ^3^H-BU99008 regional distribution binding studies in postmortem hippocampus, frontal cortex, parietal cortex, temporal cortex and cerebellum tissue from six CNs (mean age 75.1 ± 7.6 years) and six AD patients (mean age 73.1 ± 9.8 years) were performed using a single concentration of ^3^H-BU99008 (1 nM) and unlabelled BU99008 (1 μM). The graph shows the comparison of ^3^H-BU99008 specific binding (fmol/mg) in homogenates from different brain regions in CN and AD brains. Data are presented as box and whiskers plots with means of three experiments performed in triplicate for each case. * p < 0.05, *** *p* = 0.0002. *Hip- Hippocampus, Fr ctx- Frontal cortex, Par ctx- Parietal cortex, Tem ctx- Temporal cortex, Cb- Cerebellum*.

^3^H-BU99008 (1 nM) regional distribution binding was assessed in five brain regions (frontal, parietal and temporal cortices, hippocampus and cerebellum) from six CNs (mean age 75.1 ± 7.6 years) and six patients with AD (mean age 73.1 ± 9.8 years). A significantly higher ^3^H-BU99008 binding was observed in the frontal cortex (*p value* = 0.0002) and hippocampus (*p value* < 0.05) of the AD brains as compared to CN brains (Fig. 6).

### ^3^H-BU99008 and ^3^H-Deprenyl autoradiography regional binding studies in CN and sporadic AD brain sections

**Fig. 7:**
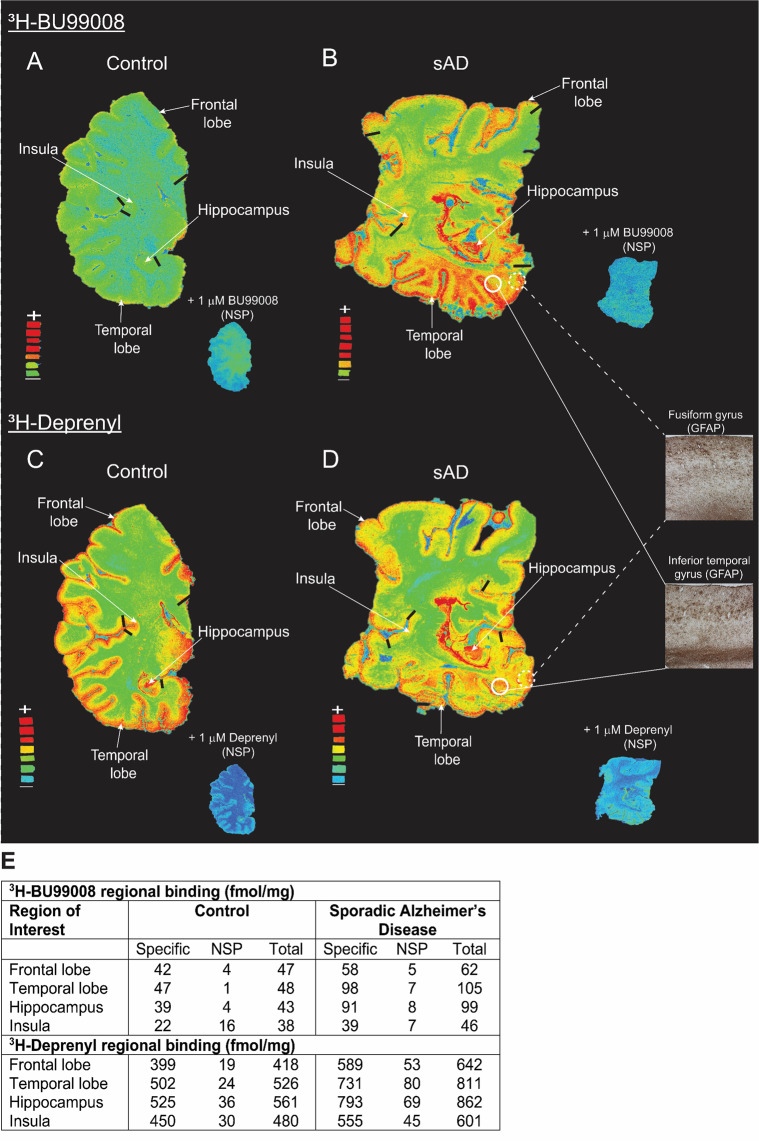
^3^H-BU99008 and ^3^H-Deprenyl autoradiography comparative studies on large frozen postmortem brain sections from CN and sporadic Alzheimer’s disease (sAD) cases. The figure shows the total binding of 1 nM ^3^H-BU99008 and 10 nM ^3^H-Deprenyl along with non-specific (NSP) binding in the presence of 1 μM unlabelled BU99008 and Deprenyl in different brain regions of CN and sAD cases, respectively. For comparison, autoradiography images of ^3^H-BU99008 (Standards: + = 3407–3580 fmol/mg, − = 58 fmol/mg) and ^3^H-Deprenyl (Standards: + = 3349–4740 fmol/mg, − = 54–77 fmol/mg) were set on the same color/brightness threshold levels of 44,461 (**A, B**) and 52,428 (**C, D**), respectively, from the raw images (16 bits: 0–65,535 (color scale)). **B** and **D** *inset*: GFAP immunostaining (astrocytes) in the temporal cortical regions of sAD case (40x magnification). **E** Regions of interest as shown in the figures were drawn manually to calculate the specific, non-specific and total binding values in fmol/mg. Frontal and temporal lobe regions were marked with dark black bars. ^3^H-Deprenyl autoradiography image for sAD case was adapted from [40]. Sporadic AD autoradiograms were rotated horizontally to be consistent (same orientation) with other images for comparison. *NSP- Non-specific binding and GFAP- Glial fibrillary acidic protein*.

^3^H-BU99008 and ^3^H-Deprenyl regional binding autoradiograms of large frozen sections from CN and AD brains are shown in Fig. 7. Visual assessment demonstrated similar regional distribution for ^3^H-BU99008 and ^3^H-Deprenyl in both the cases, with higher total binding in the sporadic AD brain as compared to CN brain (Fig. 7). ^3^H-BU99008 and ^3^H-Deprenyl both showed higher binding intensity in the temporal cortex and hippocampal regions as compared to CN brain (Fig. 7A–D). In addition, GFAP staining also demonstrated intense binding in the temporal cortical regions such as fusiform gyrus and inferior temporal gyrus of sporadic AD brain (Fig. 7B and D; ***Inset***). We performed semiquantitative analyses to further assess ^3^H-BU99008 and ^3^H-Deprenyl regional binding differences and to calculate specific, NSP and total binding in the ROI such as frontal lobe, temporal lobe, insula and hippocampus. The semiquantitative analyses results for each case are presented in Fig. 7E. The specific binding of ^3^H-BU99008 in the cortical, subcortical and hippocampal regions of the sporadic AD brain was ~1.4–2.3-fold higher as compared to the CN (Fig. 7E). For example, the specific binding of ^3^H-BU99008 in the hippocampal region of sporadic AD brain was 91 fmol/mg as compared to 39 fmol/mg in CN brain. For ^3^H-BU99008, co-incubation of sections with 1 μM unlabelled BU99008 showed ~6–15% NSP binding in sporadic Alzheimer’ disease brain ROI (7B and E), whereas in CN brain NSP binding was ~2–9% in the ROI except in the subcortical region where NSP binding was ~42% (Fig. 7A and E). In case of ^3^H-Deprenyl, the specific binding in the cortical, subcortical and hippocampal regions of sporadic AD brain was ~1.2–1.5-fold higher as compared to the CN brain (Fig. 7E). Moreover, ^3^H-Deprenyl NSP after co-incubation of sections with 1 μM unlabelled deprenyl was ~4–6 % and ~7–10% of the total binding in these ROI in CN and sporadic AD brains, respectively (Fig. 7C, D and E). We also investigated the autoradiography regional binding differences between ^3^H-BU99008 and ^3^H-Deprenyl in the *AβPParc* mutation carrier brain sections (Supplementary Fig. 1). ^3^H-BU99008 showed relatively high total binding in the ROI of *AβPParc* mutation carrier brain section as compared to CN and sporadic AD brains (compare Fig. 7A, B and E*with* Supplementary Fig. 1A and C). Whereas the total binding for ^3^H-Deprenyl in CN and *AβPParc* mutation carrier brains ROI were almost comparable except for the hippocampal region of the *AβPParc* mutation carrier brain where binding was ~1.3-fold higher (compare Fig. 7C, D and E*with* Supplementary Fig. 1B and C). In addition, ^3^H-Deprenyl also showed much higher NSP (~14–17%) as compared to ^3^H-BU99008 (~2–8%) in the *AβPParc* mutation carrier brain section (Supplementary Fig. 1).

## Discussion

The name ‘Astrocytes’ was coined by Micheal (Mihály) von Lenhossék in 1895 to represent a subtype of parenchymal glia cells [53]. Astrocytes are the main homeostatic cells in the CNS. They are highly heterogenous and can be further sub-classified into different types based on their regional distribution in the brain, for example, fibrous and velate astrocytes of the white matter and cerebellum, respectively [54]. They can undergo disease-specific modifications and complex neuropathological changes such as astrodegeneration (morphological atrophy and loss of function) and pathological remodelling (dysfunctional astroglia), that can further evolve with the disease progression, in addition to reactive astrogliosis [54]. As recent studies have put early reactive astrogliosis and glial-mediated neuroinflammation in the time course of AD pathogenesis, there is high interest in developing novel specific astrocytic-PET tracers to monitor the function of glial-neuronal network and to understand the time course of reactive astrogliosis during early-stages of disease progression. In this study, we reported for the first time the characterisation of ^3^H-BU99008 regional binding properties and its comparison with I_2_BS specific ligands (2-BFI and BU224) and deprenyl using radioligand binding studies in postmortem AD and CN brain tissues. We have also presented results of a head-to-head in vitro autoradiography comparison of ^3^H-BU99008 and ^3^H-Deprenyl binding behaviour in CN, sporadic AD and *AβPParc* mutation carrier cases using large frozen human brain sections to further evaluate the potential of BU99008 as a novel astrocytic-PET tracer for AD and other dementia disorders.

Our saturation binding studies demonstrated the presence of two-binding sites with high-and low-affinity for ^3^H-BU99008 in both CN and AD temporal cortex brain tissue homogenates with a 60-fold lower binding affinity and 5-fold higher Bmax for the major low-affinity site of ^3^H-BU99008 in AD compared to CN tissue (Bmax = 316.2 fmol/mg compared to Bmax = 58.8 fmol/mg, respectively). This observation is in agreement with previous findings using ^3^H-idazoxan that showed upregulation in the density of I_2_BS in postmortem AD brain tissue samples [50] and appears to suggest that the low-affinity BU99008 site corresponds to the I_2_BS. The difference in ^3^H-BU99008 binding affinities between the CN and AD brain tissue could be attributed to the substantial heterogeneity and complex changes in the subtypes of astrocytes in AD pathology. It is well documented that physiological and morphological properties of astrocytes can vary from region to region or within the same region in the brain and that they can respond differently via a range of activation states at both cellular and molecular levels in normal and diseased cases [12, 17, 55–58]. We also observed based on our in vivo ^11^C-DED PET studies from presymptomatic to symptomatic stages of AD [27, 29, 36, 37] and in vitro postmortem brain studies at end stage of the disease [59] (*see also Ni et al.; JAD; manuscript in press*) suggest that there are dynamic changes in DED binding in AD; increased DED binding at presymptomatic/prodromal stages, lower DED binding (at AD dementia) and then at late stage of dementia (postmortem) increase again in DED binding which most probably reflect different stage of the astrocytes as well as different forms of astrocytes (e.g. A1, A2) [12]. It is probable that similar dynamic changes in BU99008 binding could also observed at different stages of AD, however, these observations need further research and explorations. Moreover, a completely new population of astrocytes, defined as disease-associated astrocytes (DAAs) has recently been identified in the AD mouse model by Habib et al. They showed that DAAs are unique to AD and appear during the early stages while also becoming more abundant as the disease progresses [60]. Based on all these reports and speculations, it is highly likely that ^3^H-BU99008 could be targeting a completely diverse population of astrocytes in CN and AD brains hence displaying different binding affinities.

^3^H-BU99008 competition studies with different unlabelled I_2_BS specific ligands such as BU99008, 2-BFI and BU224 as well as with MAO inhibitor deprenyl revealed multiple binding sites with a wide range of binding affinities in both CN and AD temporal cortex brain tissue homogenates. We categorised these binding sites into three groups (based on their IC50 values; Fig. 3): *Super high-affinity* (SH), *High-affinity* (HA) and *Low-affinity* (LA) *binding sites*. The proportions (%) of these sites varied significantly between compounds as well as between CN and AD cases with BU99008 explicitly showing a majority of binding (~75–78%) to HA binding sites in both cases. On the contrary, similar competition studies of ^3^H-Deprenyl with unlabelled BU99008 and deprenyl demonstrated completely opposing binding behaviour for both compounds, with BU99008 exhibiting the majority of binding (~70–78%) to LA binding sites and deprenyl to HA binding sites (100%; one-site fit with IC50 value of ~20 nM) in both CN and AD cases, highlighting differences in ^3^H-BU99008 and ^3^H-Deprenyl binding behaviour. A recent study with the novel MAO B PET tracer ^18^F-SMBT also demonstrated a single binding site with a Kd value of 3.5 nM in AD brain homogenate [61], which further complement our findings that MAO B ligands can only target/detect one-site in AD brains.

An important aspect to consider while interpreting in vitro binding results is that MAO B co-expresses with I_2_BS and also possesses a distinct I_2_B site that is situated away from the active/substrate site [62, 63] and thereby possibility of ^3^H-BU99008 off-target binding to MAO B is very likely. However, recently published in vivo blocking studies with MAO B and I_2_BS inhibitors in rhesus monkeys and healthy CNs demonstrated a high selectivity and specificity of radiolabeled BU99008 for astrocytic I_2_BS [43, 51]. Both studies showed that the brain uptake of ^11^C-BU99008 was not affected by blocking with MAO B inhibitors whereas pretreatment with selective I_2_BS inhibitor BU224 significantly reduced the ^11^C-BU99008 signal throughout the rhesus brain in a dose-dependent manner. In case of human brain, pretreatment with the mixed I_2_BS/α2-adrenoceptor inhibitor idazoxan also reduced ^11^C-BU99008 uptake throughout the brain with an average block of 60% across all regions at the highest dose of 80 mg. As the blockade was not dose-dependent, authors suggested that this could be due to biphasic blockade by idazoxan. We also demonstrated similar properties for ^3^H-BU99008 using competition studies, where binding of BU99008 to HA binding sites in both CN and AD cases was not affected by the increasing concentrations of MAO B inhibitors deprenyl and safinamide. However, binding of BU99008 to the SH binding sites in CN was completely blocked in the presence of deprenyl or safinamide along with a decrease in ^3^H-BU99008 total binding suggesting two possibilities: First, that the SH binding site might represent the MAO B binding site. Second, that MAO B inhibitors might interact with BU99008 and subsequently interfere with BU99008 binding, with the extent of interference depending on the mode of binding of these inhibitors on MAO B (see Edmondson et al., 2009, for details regarding MAO B inhibitors binding mechanisms). These observations are an incentive to perform further explorative mechanistic and structural based studies.

In this study, we also compared the regional distribution of ^3^H-BU99008 binding in CN and AD brain tissue homogenates and demonstrated significantly more extensive ^3^H-BU99008 binding in the frontal cortex (*p* = 0.0002) and hippocampus (*p* < 0.05) of the AD brains as compared to CN brains. We observed lowest binding for ^3^H-BU99008 in the cerebellum which was expected based on the recently published ^11^C-BU99008 human in vivo studies where tracer uptake in cerebellum was also low [43]. Even though we also observed differences in ^3^H-BU99008 binding between CN and AD brains in the temporal cortex, they were not as significant as observed in our saturation studies. One of the reasons could be the differences in the experimental setup, as regional binding studies were normally performed with a single concentration of ^3^H-BU99008 (1 nM), whereas a much wider concentration range was used in saturation binding studies (0–35 nM). The possibility of detecting all the binding sites (with high-, intermediate- and low-affinity) is much higher with a broader concentration range. Moreover, regional binding studies were performed on a larger number of cases (*n* = 6) whereas only one case was used for the saturation binding studies. This also points towards possible case-by-case variability and reintroduce the concept of astrocyte heterogeneity back into the picture as discussed above. Regardless of this, our findings are in agreement with earlier postmortem AD brain tissue studies, where increased reactive astrogliosis was observed in the cortical and hippocampal regions of brains with high amyloid-β or tau burdens [20, 39, 64, 65].

Autoradiography comparative studies suggested similar regional binding patterns for both ^3^H-BU99008 and ^3^H-Deprenyl in sporadic AD, *AβPParc* mutation carrier and CN brains. However, a difference was observed in the numeric binding intensity of the two tracers which could be either due to different concentrations of tracer used for the experiments (10 nM for ^3^H-Deprenyl vs. 1 nM for ^3^H-BU99008) or higher NSP binding in case of ^3^H-Deprenyl. The binding distribution of ^3^H-BU99008 followed the rank order: hippocampus and cortex > subcortical regions. This observation was in line with our ^3^H-BU99008 brain homogenate regional binding findings and previous human brain autoradiography studies showing the highest I_2_BS densities in hippocampal regions (dentate gyrus) and more moderate densities in cortical regions [66, 67]. In addition, the ^3^H-BU99008 binding distribution correlated very well with the increased GFAP immunoreactive levels in the temporal cortical areas. It is important to mention here that in vivo ^11^C-BU99008 human PET-studies also highlighted some off-target bindings in striatum and other subcortical areas with low I_2_Bs density [43]. However, this need to be further confirmed in large sample size as they suggested that higher binding in these areas should be carefully interpreted due to large test-retest variability. We also observed a higher binding for ^3^H-BU99008 in the *AβPParc* mutation carrier brains as compared to sporadic AD brains this could be due to pathological differences between the two cases as the *AβPParc* mutation has been shown to promote the formation of amyloid-β oligomers and protofibrils [68]. On the other hand, the binding for ^3^H-Deprenyl was comparable between the CN and the *AβPParc* mutation carrier brains. These observations further highlight possible differences in binding properties between ^3^H-BU99008 and ^3^H-Deprenyl (as also observed in our competition studies) and indicate that their targets are most likely somewhat different. It is possible that ^3^H-BU99008 and ^3^H-Deprenyl are targeting a different subtype of reactive astrocyte or a specific reactive state, not all reactive astrocytes. However, it is very important to keep the fact in mind that large frozen hemisphere sections are rare material and the sections used in this study did not originate from exactly the same coronal anatomical level; therefore the results of direct comparison between the cases should be interpreted cautiously. To summarise, our findings suggest that ^3^H-BU99008 could detect I_2_BS expressing reactive astrocytes with good selectivity and specificity, as also recently demonstrated by an in vivo ^11^C-BU99008 PET-study in Parkinson’s disease patients [69]. However, one limitation of this study is that the binding studies were performed on a small number of brains and it would be interesting to further explore the ^3^H-BU99008 binding characteristics in larger sample sizes.

Overall, this study reports, for the first time, the presence of multiple binding sites (SH, HA and LA) for ^3^H-BU99008, I_2_BS-specific ligands and deprenyl in CN and AD brains and demonstrate that ^3^H-BU99008 could possibly detect/target reactive astrogliosis in AD brains. Based on our competition and regional binding studies and upon comparison with the MAO-B inhibitor deprenyl, we conclude that ^3^H-BU99008 binds specifically to HA binding sites and its binding behaviour and properties appear to be different from those of ^3^H-Deprenyl in CN, and sporadic AD brains. Even though studies have established reactive astrogliosis as one of the hallmarks of the AD brains, their role in the pathogenesis of the disease is still a topic of debate. Further in vivo and in vitro studies in large numbers of CN and sporadic and autosomal dominant AD cases are needed to establish the reliability of BU99008 as a specific PET-tracer and I_2_BS as a surrogate marker for reactive astrogliosis. This will contribute to a better understanding of the time course and pathophysiological role of reactive astrogliosis in AD.

## Supplementary information

Supplementary Figure 1

## Data Availability

All the data supporting the findings of this study are presented in the paper. Raw data for this study may be available from the corresponding author upon reasonable request.
