# Supplementary material for: Astroglial tracer BU99008 detects multiple binding sites in Alzheimer’s disease brain
Source: Mol Psychiatry. 2021 Apr 23;26(10):5833–47. doi: 10.1038/s41380-021-01101-5 (PMC8758481; doi:10.1038/s41380-021-01101-5)
Supplement: Supplementary file 1 — Supplementary Figure 1 [file 41380_2021_1101_MOESM1_ESM.pdf]

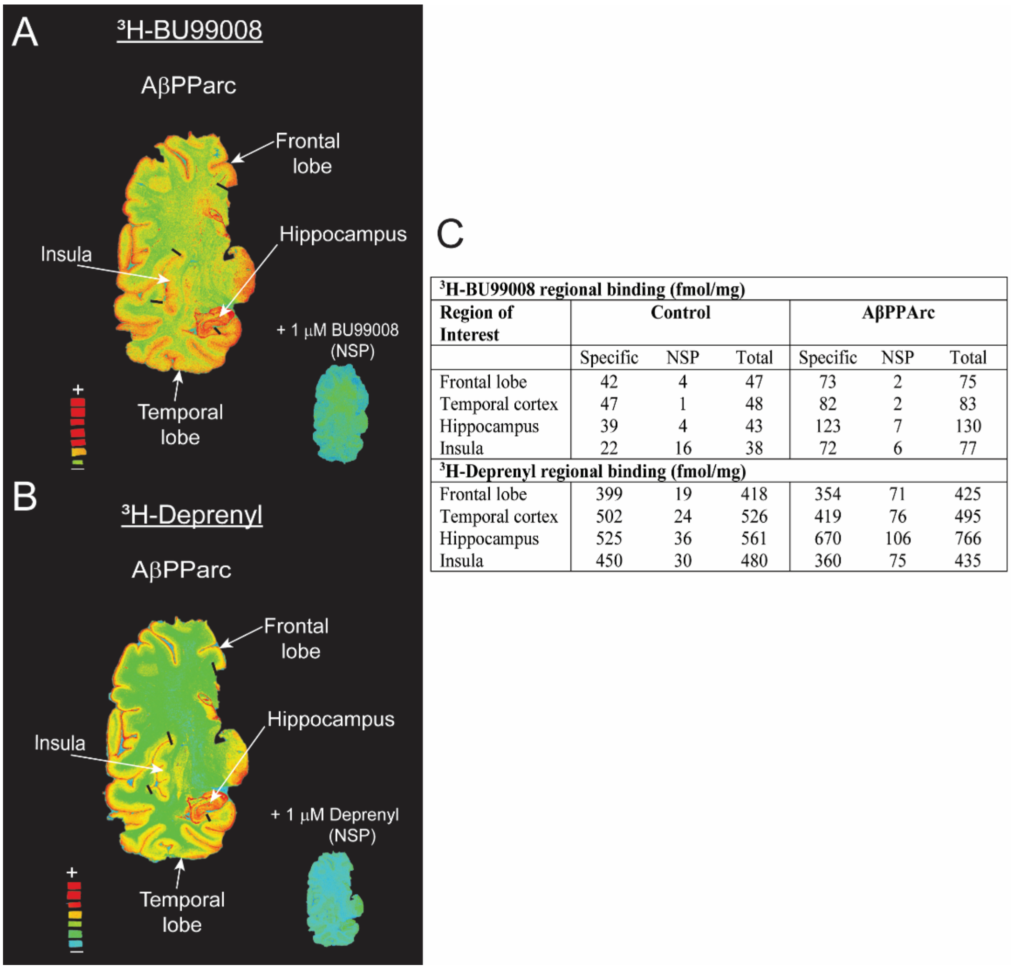

C

**^{3}H-BU99008 regional binding (fmol/mg)**						
**Region of Interest**	**Control**			**AβPPArc**		
	Specific	NSP	Total	Specific	NSP	Total
Frontal lobe	42	4	47	73	2	75
Temporal cortex	47	1	48	82	2	83
Hippocampus	39	4	43	123	7	130
Insula	22	16	38	72	6	77
**^{3}H-Deprenyl regional binding (fmol/mg)**						
Frontal lobe	399	19	418	354	71	425
Temporal cortex	502	24	526	419	76	495
Hippocampus	525	36	561	670	106	766
Insula	450	30	480	360	75	435

Supplementary Fig. 1. ^{3}H-BU99008 and ^{3}H-Deprenyl autoradiography comparative studies on large frozen postmortem brain sections from Arctic amyloid-β protein precursor (*AβPPArc*) case.

The figure shows the total binding of 1 nM ^{3}H-BU99008 and 10 nM ^{3}H-Deprenyl along with non-specific (NSP) binding in the presence of 1 μM unlabelled BU99008 and Deprenyl in different brain regions of AβPPArc case. Autoradiography images of ^{3}H-BU99008 (Standards: + = 3407-3580 fmol/mg, – = 58 fmol/mg) and ^{3}H-Deprenyl (Standards: + = 3349-4740 fmol/mg, – = 54-77 fmol/mg) were set on the color/brightness threshold levels of 44,461 (**A**) and 52,428 (**B**), respectively, from the raw images (16 bits: 0-65,535 (color scale)) for comparison with **Fig. 7**. (**C**) Regions of interest as shown in the figures were drawn manually to calculate the specific, non-specific and total binding values in fmol/mg. The regional binding

values in different region of interest for both control and *AβPPArc* cases for comparison are presented here. Frontal and temporal lobe regions were marked with dark black bars. ^{3}H-Deprenyl autoradiography image for *AβPPArc* case was adapted from Lemoine et al. 2020 (37). *NSP- Non-specific binding*
